# Acetaminophen: A hazard to immunotherapy

**DOI:** 10.1016/j.amsu.2022.104272

**Published:** 2022-07-31

**Authors:** Najeebullah, Maria Ameer Ali, Rida Naveed, Govinda Khatri, Priya, Mohammad Mehedi Hasan

**Affiliations:** a Dow University of Health Sciences, Karachi, Pakistan; b Department of Biochemistry and Molecular Biology, Faculty of Life Science, Mawlana Bhashani Science and Technology University, Tangail, Bangladesh

Acetaminophen (APAP) also known as paracetamol and Tylenol is a widely used anti-analgesic and antipyretic that has been used in therapeutic practice for over a century. It's commonly used to treat headaches, toothaches, osteoarthritic pain, and even post-operative pain [1]. For a drug that is so widely used, the exact mechanism of action is still a mystery. However, different studies have led to the widespread belief that acetaminophen acts on the central serotonergic system to provide analgesic effects. Pickering et al. concluded through a clinical trial that co-administration of hydroxytryptamine type 3 antagonists such as tropisetron (5 mg) and granisetron (3 mg) with acetaminophen (1 mg) blocks the anti-analgesic impact of acetaminophen on the central serotonergic system [2]. Acetaminophen has been shown to penetrate the blood-brain barrier in humans, according to research [3]. As a result, it further strengthens the idea that APAP has a relationship to the serotonergic system. When taken as prescribed, acetaminophen is well tolerated; nevertheless, hypersensitivity, nephrotoxicity, and pancytopenia are some of the side effects of orally or rectally administered APAP. Nausea, vomiting, constipation, stomach pain, and pruritus are all possible side effects of intravenous APAP [4]. Acetaminophen (APAP) overdose is linked to hepatotoxicity and acute liver failure [5]. The FDA-approved standard antidote for APAP overdose is *N*-acetyl cysteine. According to recent findings, fomepizole could be used as an additional antidote for APAP poisoning [6].

A recent study presented in the annual meeting of the American Society of Clinical Oncology and later on published in the Journal of Annals of Oncology in May 2022 raised new and more serious concerns — it revealed that cancer patients who take acetaminophen as premedication have a much-reduced response to immunotherapy. Immunotherapy works by blocking immune checkpoints, which enable immune cells to fight cancer more effectively [7]. In a study, 600 patients with advanced cancer were given acetaminophen before starting immunotherapy, and the response of immune checkpoint inhibitors was assessed after the immunotherapy was started. The response of immune checkpoint inhibitors was dramatically reduced, implying that acetaminophen (APAP) has immunosuppressive effects [8]. Acetaminophen (APAP) is frequently used to manage cancer-associated pain. A recent study looked at the effects of acetaminophen on cancer patients who were taking immune check inhibitors (ICB) [8]. T cells can destroy tumor cells by blocking the binding of checkpoints which enable immune cells to fight cancer more effectively. T cells can destroy tumor cells by blocking the binding of checkpoint proteins like PDL1 to PD1 with immune checkpoint blockers [7]. The effects of acetaminophen on a preclinical tumor model (MC 38) revealed that it significantly reduced the potency of ICB [9]. According to another study, acetaminophen therapy followed by interferon-gamma (20 ng/ml) for 2 h was linked to immune checkpoint PDL1 expression stimulation [10]. Various other researchers have struggled to explain why acetaminophen suppresses the immune system. According to Neil et al. Aspirin and acetaminophen were tightly related to the reduction of the serum neutralizing antibody response towards the study challenge virus, Rhinovirus type 2. Acetaminophen and aspirin both enhance RV2 viral shedding considerably. Ibuprofen had a weaker effect on antibody levels, and it did not differ significantly from placebo [11]. Another identical research concluded that acetaminophen helps increase the duration of varicella infections in children, supporting the idea that acetaminophen reduces the human immune system [12] Paracetamol is given prophylactically before or shortly after the vaccination to reduce the pain and fever associated with vaccinations. However, new research has revealed that such prophylactic treatment may be interfering with the development of an immune response to vaccine antigens [13]. When acetaminophen is administered prophylactically, it diminishes the response of Hepatitis B Antibodies (Anti-HBs) to Hepatitis B vaccination in adults. According to a study Anti-HBs levels in the group receiving therapeutic paracetamol were found to be comparable to those in the control group a month after the second booster was administered. However, Anti HBs levels were noticeably lower in. the paracetamol group is given as a preventive [14]. It was also revealed from Animal trials that acetaminophen (APAP) decreases the antibody response towards Sheep red blood cells (SRBC) in both fed and fasted mice indicating acetaminophen has an immunosuppressive impact on humoral immunity [15].

In light of these studies, the question still stands: Is acetaminophen safe to be considered as a premedication drug before initiating immunotherapy in patients with advanced cancer? The aforementioned studies are of chief importance and it is concerning because acetaminophen is still the first-line strategy to manage mild-to-moderate pain among patients with advanced cancer. The data provided highlights something that could dramatically change the way we manage our patients who are on immune check inhibitors. Health workers should use acetaminophen with care in patients who are on immunotherapy.

## Ethical approval

NA.

## Sources of funding

NA.

## Author contribution

All authors meet the inclusion criteria, and all authors read and approved the final version of the manuscript.

## Trail registry number

1. Name of the registry: NA.

2. Unique Identifying number or registration ID: NA.

3. Hyperlink to your specific registration (must be publicly accessible and will be checked): NA.

## Guarantor

Mohammad Mehedi Hasan.

Department of Biochemistry and Molecular Biology, Faculty of Life Science, Mawlana Bhashani Science and Technology University, Tangail, 1902, Bangladesh.

Email: mehedi.bmb.mbstu@gmail.com.

## Annals of medicine and surgery

The following information is required for submission. Please note that failure to respond to these questions/statements will mean your submission will be returned. If you have nothing to declare in any of these categories then this should be stated.

## Consent

NA.

## Declaration of competing interest

NA.
